# The co-occurrence of peri-prosthetic fracture and infection: a scoping review on current diagnostic principles, treatment and outcome

**DOI:** 10.5194/jbji-11-635-2026

**Published:** 2026-10-14

**Authors:** Noémie Reinert, Laura Bessems, Martin Clauss, Nader Maai, Jolien Onsea, Georges Vles, Karl Stoffel, Willem-Jan Metsemakers, Mario Morgenstern

**Affiliations:** 1 Center for Musculoskeletal Infections, University Hospital of Basel, Basel, Switzerland; 2 Department of Orthopedic and Trauma Surgery, University Hospital Basel, Basel, Switzerland; 3 Department of Trauma Surgery, University Hospitals Leuven, Leuven, Belgium; 4 Department of Development and Regeneration, KU Leuven, Leuven, Belgium; 5 Department of Orthopedic Surgery, University Hospitals Leuven, Leuven, Belgium

## Abstract

**Background**: The co-occurrence of periprosthetic fractures and infection, referred to as infected periprosthetic fracture (iPPF), represents a highly burdensome complication in orthopedic surgery. Management is challenging since not rarely treatment principles of prosthetic joint infections and fracture-related infections have to be combined. The aim of this scoping review was to map the current literature on management and outcome of iPPFs. **Methods**: A scoping review was conducted in accordance with PRISMA-ScR guidelines. Five major databases were systematically searched. Studies reporting on diagnostic principles and/or treatment strategies for iPPF around the hip and knee were included. Data were mapped regarding study design, diagnostic approaches, surgical strategies, and outcome. **Results**: A total of 32 studies comprising 273 patients with iPPFs were included; 8 addressed diagnostic strategies, and 24 reported on treatment and outcome. Diagnostic studies demonstrated limited reliability of serum inflammatory markers, and although synovial fluid analysis appeared most informative, optimal thresholds remain uncertain. Most treatment studies were small and heterogeneous. A staged surgical approach, predominantly two-stage revision, was reported in 83 % of the included studies. Across nine studies reporting outcomes, mean infection eradication and recurrence rates were 76.1 % and 22.3 %, respectively. The majority of the studies (58.3 %) described that additional surgeries were needed to cure infection. Fracture healing outcomes were inconsistently reported (bone healing in 4 out of 9 studies with 53/58 fractures, mean 91.4 %). **Conclusion**: This scoping review demonstrates that management of iPPFs remains highly challenging, with high rates of recurrent infection and unplanned revision surgery. However, the reported quantitative outcome data represent descriptive summaries derived from heterogeneous, predominantly small observational studies and case series and should not be interpreted as definitive benchmarks for clinical outcomes. The current evidence base remains limited, highlighting the need for standardized diagnostic criteria, uniform outcome reporting, and well-designed prospective multicenter studies.

## 1 Introduction

Periprosthetic fractures (PPFs) are known to be devastating complications after joint arthroplasty and are associated with considerable morbidity and mortality. With an aging population and a continuous rise in arthroplasty procedures, the incidence of PPFs is increasing and represents a growing clinical and socioeconomic challenge (Rupp et al., 2022). These injuries are associated with high complication rates such as instability, nonunion or infection (El Khassawna et al., 2021; Moreta et al., 2021), and high mortality rate up to 60 % (Walter et al., 2024). In the recent literature, high infection rates ranging from 5.6 % to 16.1 % for PPFs have been reported (Luyckx et al., 2022; Miettinen et al., 2023; Preston et al., 2015). Notably, even infection without fracture (isolated prosthetic joint infection, PJI) carries substantial mortality – 11 % after infected total hip arthroplasty (THA) and 13 % after infected total knee arthroplasty (TKA) (Ramos et al., 2025, 2026), which is comparable to common cancers. The co-occurrence of both PPF and an infection, hereafter referred to as an infected periprosthetic fracture (iPPF), represents a hybrid entity combining aspects of both PJIs and fracture-related infections (FRIs). Consequently, iPPFs require clinicians to be familiar with principles of both PJI and FRI management. However, these objectives may conflict. In chronic PJIs, treatment often involves a two-stage procedure with an antibiotic-loaded spacer and an implant-free interval. In contrast, successful FRI management requires stable fixation to promote bone healing and infection eradication. The management of iPPFs therefore necessitates reconciling two potentially contradictory principles: infection eradication through implant removal and maintenance of mechanical stability (Müller et al., 2019). The diagnosis of iPPF is equally demanding, since inflammatory markers are not reliable and of poor predictive value in the history of recent trauma (Chevillotte et al., 2009).

Since existing evidence of iPPFs is sparse and heterogeneous, the objective of this scoping review is twofold: (1) to systematically map the published treatment concepts and reported outcomes for iPPFs and (2) to provide an overview of current diagnostic principles.

## 2 Methods

### 2.1 Reporting guidelines

This scoping review was conducted in accordance with the PRISMA-ScR guidelines (Tricco et al., 2018), and the protocol has been pre-registered on the Open Science Framework platform (Reinert et al., 2025b).

### 2.2 Literature search strategy

A comprehensive search of the biomedical databases – PubMed (including MEDLINE), Embase, Web of Science Core Collection, Scopus, and CENTRAL (via Cochrane Library) – was conducted on 8 January 2026. The strategy was developed in collaboration with biomedical reference librarians. The search strategy combined MeSH/Emtree terms and free-text keywords representing the concepts “periprosthetic fracture” and “infection”. After finalizing the search strategy in Pubmed, the framework was extended to the other included databases and checked for consistency. Search limits were restricted to the English language. Gray literature was taken into account for a more continuous screening process with the inclusion of Clinicaltrials.gov and the International Clinical Trials Registry Platform (ICTRP).

### 2.3 Study eligibility and selection

All retrieved records were imported into Endnote Version 21 (Clarivate, USA), and deduplication was performed. Using the Rayyan platform, the primary studies were screened by two independent reviewers (Noemie Reinert and Laura Bessems). In the case of disagreement, a third reviewer was consulted (JO). Titles and abstracts were screened with respect to the predefined eligibility criteria. The full text of the relevant articles was then matched against our inclusion and exclusion criteria, and any reasons for in- or exclusion were documented.

The Joanna Briggs Institute PCC framework for scoping reviews was applied (Pollock et al., 2023):

- *population* – patients with a periprosthetic fracture and infection;
- *concept* – treatment algorithm and outcome, diagnostic principles;
- *context* – hip joint (acetabulum, proximal femur, shaft, distal femur) and knee joint (proximal tibia and shaft).

Eligible study designs included observational studies and case reports. Exclusion criteria were other anatomical locations than hip and knee joint, animal studies, conference abstracts, narrative reviews without original patient data, editorials/letters, and fractures due to malignant/benign tumors or metabolic bone diseases.

### 2.4 Data extraction and presentation

The study selection process was summarized using a PRISMA flow diagram (Fig. 1). Included studies were grouped into diagnostic and treatment papers. Extraction of relevant data was recorded for the following key information: author, title and year of the publication, the study design and sample size, fracture classification, infection definition, surgical treatment concepts addressing iPPF, follow-up intervals, infection outcome, and bone healing. For outcome analysis only studies that include a minimum of five patients were considered.

**Figure 1:**
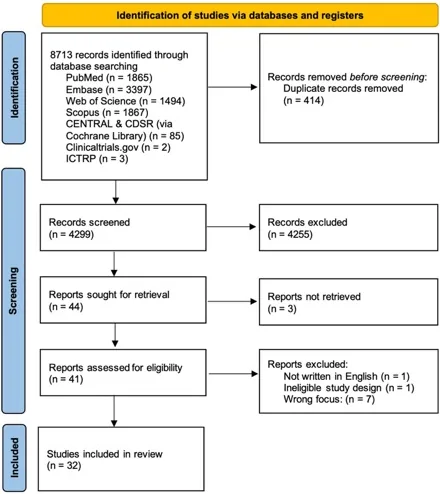
PRISMA flow diagram.

### 2.5 Infection scenarios

IPPFs were categorized into two predefined scenarios.

- *Scenario F-I.* A PPF is treated with fracture fixation or revision arthroplasty, followed by an infection. Revision arthroplasty is defined by removing and replacing or exchanging one or more prosthetic components due to complication (fracture), e.g. femoral stem revision in a Vancouver B2 fracture (Liebs et al., 2015).
- *Scenario I-F.* A PPF occurs in the setting of an already infected prosthesis. This includes fractures as a result of implant loosening with proof of infection in the PPF revision surgery (scenario I-F-A) or fractures during treatment of a confirmed PJI (during explantation, re-implantation, exchange, or between stages) (scenario I-F-B).

## 3 Results

After applying the predefined inclusion and exclusion criteria to 4299 retrieved articles, a total of 32 studies reporting on iPPFs were included for analysis and synthesis (Fig. 1). The eligible articles comprising in total 273 patients included 8 diagnostic papers (Table 1) and 24 treatment papers (Table 2).

**Table 1:** Overview of diagnostic papers ( n=8 ).

Author (year)	Study design	PPFs/iPPFs ( n )	Infection definition	Fracture classification	Localization	Diagnostic procedure
Barberis et al. (2024)	Retrospective cohort study	220/15	MSIS/ICM	Vancouver, Rorabeck	Hip and knee	Intraoperative cultures (unexpected positive cultures), serum CRP
Chevillotte et al. (2009)	Retrospective cohort study	204/21	Two or more positive intraoperative tissue samples or positive intra-articular aspiration cultures	Vancouver	Hip	Serum inflammatory markers (CRP, ESR, WBC)
Lazic et al. (2022)	Prospective cohort study	30/14	EBJIS consensus definition	Vancouver, Rorabeck, and Paprosky	Hip and knee	Synovial calprotectin, serum CRP, synovial WBC, and % PMN
Luyckx et al. (2022)	Retrospective cohort study	24/2	MSIS/ICM	Vancouver	Hi	Intraoperative frozen section histopathology ( ≥5 PMN/HPF)
Muñoz-Mahamud et al. (2013)	Retrospective cohort study	11/2	Growth of the same microorganism on at least two cultures of deep samples of periprosthetic tissue and/or presence of pus surrounding the prosthesis	Vancouver	Hip	Histopathology ( ≥5 PMN/HPF), intraoperative cultures
Preston et al. (2015)	Retrospective cohort study	27/2	MSIS/ICM	Vancouver, Rorabeck	Hip and knee	Preoperative synovial fluid nucleated cell count
Shah et al. (2016)	Retrospective cohort study	121/4	MSIS/ICM	No specific classification	Hip and knee	Serum markers (CRP, ESR, WBC) and synovial WBC and % PMN
Van Den Kieboom et al. (2021)	Retrospective cohort study	144/41	MSIS/ICM	No specific classification	Hip and knee	Serum markers (CRP, ESR) and synovial WBC and % PMN

MSIS/ICM: Musculoskeletal Infection Society/International Consensus Meeting; CRP: C-reactive protein; ESR: erythrocyte sedimentation rate; WBC: white blood cell count; EBJIS: European Bone and Joint Infection Society; PMN/HPF: polymorphonuclear cells per high-power field.

**Table 2:** Overview of therapeutic papers ( n=24 ).

Author (year)	Study design	iPPFs ( n )	Infection scenario	Fracture classification	Localization	Main surgical strategy	Chronicity
Baker et al. (2012)	Case report	1	F-I	Vancouver	Hip	DAIR	2 weeks after PPF fixation
Bonacker et al. (2018)	Case report	1	F-I	Felix	Knee	Failed DAIR, conversion to two-stage revision surgery	Not reported
Bottner et al. (2005)	Case series	2	F-I	No specific classification	Hip and knee	Two-stage revision surgery	Not reported
Cabral (2012)	Retrospective observational study	3	F-I	Modified Vancouver (Huc) classification	Hip	Two-stage revision surgery	Not reported
Duong et al. (2025)	Retrospective observational study	12	Not clear	Vancouver	Hip	Two-stage revision surgery	9.6 months (range 2 to 28 months) after arthroplasty
Faggiani et al. (2025)	Case series	9	F-I	Vancouver, Rorabeck, and Felix	Hip and knee	Implant removal, one-stage and two-stage revision surgery	304 d (range 16–1551 d) after PPF surgery
Font-Vizcarra et al. (2014)	Case report	1	F-I	Felix	Knee	Failed DAIR, conversion to two-stage revision surgery	4 weeks after PPF surgery
Hagen et al. (2013)	Case report	1	F-I	No specific classification	Hip and knee	Two-stage revision surgery	2 months after PPF fixation
Hao et al. (2023)	Case report	1	I-F	Rorabeck and Taylor	Knee	Two-stage revision surgery	PPF 3 months after PJI diagnosis
Jaschke et al. (2025)	Retrospective cohort	46	I-F	Vancouver and Paprovsky	Hip	Two-stage revision surgery	During explantation or interstage
Kamath et al. (2012)	Case report	1	I-F	No specific classification	Hip and knee	Two-stage revision surgery	Not reported
Karczewski et al. (2022)	Retrospective cohort	9	I-F	AO classification	Hip and knee	Two-stage revision surgery	Before/during explantation or reimplantation
Keller et al. (2022)	Retrospective observational study	10	F-I	No specific classification	Hip and knee	Two-stage revision surgery	Not reported
Kim et al. (2017)	Case report	1	I-F	Vancouver	Hip	Two-stage revision surgery	Not reported

**Table 2:** Continued.

Author (year)	Study design	iPPFs ( n )	Infection scenario	Fracture classification	Localization	Main surgical strategy	Chronicity
Konan et al. (2011)	Retrospective observational study	17	I-F	Vancouver	Hip	One- or two-stage revision surgery	Not reported
Kraus et al. (2025)	Case report	1	F-I	Vancouver	Hip	Two-stage revision surgery	11 d after PPF revision surgery
Maceroli et al. (2019)	Case report	1	I-F	No specific classification	Hip	Two-stage revision surgery	Not reported; during staged PJI treatment
Matsuno et al. (2023)	Retrospective observational study	12	F-I	Vancouver	Hip	Implant removal, DAIR, two-stage revision surgery	Not reported
Müller et al. (2019)	Prospective observational study	8	Mixed	Vancouver	Hip	Two-stage revision surgery (three-step algorithm)	Mixed chronicity; intervals not reported
Nasser et al. (2020)	Case report	1	I-F	Vancouver	Hip	Failed DAIR, conversion to two-stage revision surgery	∼10 years before PPF
Pang et al. (2015)	Case report	1	F-I	Rorabeck	Knee	Two-stage revision surgery	Not reported
Sassoon et al. (2014)	Retrospective observational study	21	I-F	No specific classification	Knee	Two-stage revision surgery	Not reported; during resection or reimplantation
Schwab et al. (2003)	Case report	1	I-F	No specific classification	Hip	Two-stage revision surgery	Not reported
Sherman et al. (2008)	Case report	1	F-I	No specific classification	Hip	Two-stage revision surgery	Not reported

I: infection; F: fracture; PPF: periprosthetic fracture; PJI: periprosthetic joint infection. For F-I cases, timing refers to the interval between PPF treatment and infection onset or diagnosis. For I-F cases, the chronicity of the pre-existing infection and/or the timing of fracture occurrence within the PJI treatment pathway is reported.

### 3.1 Diagnostic papers

The 8 studies included 111 patients with an iPPF and 670 patients with a PPF but no infection (control group). Across included studies, diagnostic evaluation for concomitant infection employed multiple approaches: serum inflammatory markers (C-reactive protein in eight studies), synovial fluid analysis including nucleated cell counts (four studies), intraoperative frozen section histopathology (two studies), and microbiological culture (standard in all studies) (Table 1) (Barberis et al., 2024; Chevillotte et al., 2009; Lazic et al., 2022; Luyckx et al., 2022; Muñoz-Mahamud et al., 2013; Preston et al., 2015; Shah et al., 2016; Van Den Kieboom et al., 2021). In six of the eight studies, infection was defined according to established criteria: five studies applied the Musculoskeletal Infection Society/International Consensus Meeting (MSIS/ICM) definition (Parvizi and Gehrke, 2014), and one study used the European Bone and Joint Infection Society (EBJIS) definition (McNally et al., 2021). In two studies no specific definition was mentioned. The Vancouver classification was the most commonly used fracture classification system (six out of eight studies), followed by the Rorabeck classification (Rorabeck and Taylor, 1999; Schopper et al., 2022). Data on the retrieved pathogens and used local antibiotics are summarized in Appendix A.

### 3.2 Treatment papers

An overview of the included 24 treatment papers comprising 162 iPPF cases is presented in Table 2 (Baker et al., 2012; Bonacker et al., 2018; Bottner et al., 2005; Cabral, 2012; Duong et al., 2025; Faggiani et al., 2025; Font-Vizcarra et al., 2014; Hagen et al., 2013; Hao et al., 2023; Jaschke et al., 2025; Kamath et al., 2012; Karczewski et al., 2022; Keller et al., 2022; Kim et al., 2017; Konan et al., 2011; Kraus et al., 2025; Maceroli et al., 2019; Matsuno et al., 2023; Müller et al., 2019; Nasser et al., 2020; Pang et al., 2015; Sassoon et al., 2014; Schwab et al., 2003; Sherman et al., 2008).

In the majority of the included studies (83.3 %; 20/24 with 125 cases) a two-stage procedure was described to treat the iPPF. One-stage exchange was only mentioned in 2 studies including 14 cases (Faggiani et al., 2025; Konan et al., 2011). In two studies a DAIR procedure was applied (Baker et al., 2012; Matsuno et al., 2023), and in three studies (each including three cases) a conversion from a DAIR to a two-stage revision was necessary due to treatment failure (Bonacker et al., 2018; Font-Vizcarra et al., 2014; Nasser et al., 2020). The majority of the studies (58.3 %; 14/24) reported that additional unplanned surgical procedures, beyond the initially intended treatment strategy (DAIR, one-stage exchange, or two-stage exchange), were required to achieve infection eradication.

Outcomes were analyzed across 9 studies, including a total of 146 cases (Table 3) (Duong et al., 2025; Faggiani et al., 2025; Jaschke et al., 2025; Karczewski et al., 2022; Keller et al., 2022; Konan et al., 2011; Matsuno et al., 2023; Müller et al., 2019; Sassoon et al., 2014). Infection eradication rate ranged from 58.3 % to 91.7 %, with a mean infection eradication rate of 76.1 % (99/130). Infection recurrence rate ranged from 8.3 % to 29.2 %, with a mean recurrence rate of 22.3 % (29/130). Bone healing outcome was reported only by 4 studies including 58 cases and ranged from 62.5 % to 100 % (mean 91.4 %; 53/58).

**Table 3:** Infection and radiological outcomes in selected treatment studies ( n=9 ).

Author (year)	Infection	Recurrence	Bone healing	Follow up
	eradication	( n (%))	( n (%))	(mean)
	( n (%))			
Duong et al. (2025)	11/12 (91.7 %)	1/12 (8.3 %)	Not reported	9.5 months
Faggiani et al. (2025)^*^	Not reported^*^	Not reported^*^	Not reported^*^	12 months
Jaschke et al. (2025)	29/41 (70.7 %)	12/41 (29.2 %)	Not reported	66 months
Karczewski et al. (2022)	7/9 (77.8 %)	2/9 (22.2 %)	Not reported	24 months
Keller et al. (2022)	8/10 (80.0 %)	2/10 (20.0 %)	10/10 (100 %)	21.4 months
Konan et al. (2011)	14/17 (82.3 %)	3/17 (17.6 %)	17/17 (100.0 %)	38 months
Matsuno et al. (2023)	7/12 (58.3 %)	3/12 (25.0 %)	Not reported	20 months
Müller et al. (2019)	7/8 (87.5 %)	1/8 (12.5 %)	5/8 (62.5 %)	34 months
Sassoon et al. (2014)	16/21 (76.2 %)	5/21 (23.8 %)	21/23 (91.0 %)	56 months

^*^ Included for provided data on follow-up and (additional) surgeries.

## 4 Discussion

IPPFs should be regarded as a hybrid clinical entity that combines characteristics and principles of FRIs and PJIs, and therefore their management must simultaneously address infection eradication, implant stability, and fracture healing.

This scoping review, which includes 32 studies (8 diagnostic and 24 treatment studies) with 273 patients with an iPPF in total, reveals that current evidence is mainly based on small-scale studies and case reports. Evidence of diagnostic strategies is still scarce, and no standardized treatment algorithm exists. Correspondingly, there is limited outcome reporting for iPPFs: infection outcomes were reported for only 130 cases and bone healing for solely 58 cases.

### 4.1 Diagnostic challenges in iPPFs

Although infection occurs in a relatively high proportion of PPFs, its diagnosis remains challenging as fracture-related inflammation interferes with the accuracy of standard diagnostic tests, and, to date, the current PJI definitions are not validated in the setting of iPPF (McNally et al., 2021). In addition, the reviewed literature demonstrates considerable heterogeneity in the infection definitions. Most included studies applied diagnostic criteria developed for PJI, particularly the MSIS/ICM or EBJIS definitions, whereas the FRI consensus definition was rarely applied, likely reflecting the historical predominance of arthroplasty-focused research. However, as iPPFs represent a hybrid pathology, neither the PJI nor the FRI diagnostic framework alone adequately captures the full spectrum of disease. A diagnostic strategy applying established PJI and FRI definitions in parallel should therefore be adopted, whereas joint involvement determines which diagnostic pathway is most relevant. Since infection may involve the prosthetic joint, the fracture site, or both simultaneously, integrating both diagnostic frameworks enables a more comprehensive assessment of infection extent and provides a stronger basis for treatment planning.

The reviewed studies consistently demonstrate considerable diagnostic limitations and underline that no single parameter is sufficiently reliable on its own. Therefore, the interpretation of laboratory, synovial, and intraoperative findings must always account for the coexistence of trauma (Chevillotte et al., 2009; Luyckx et al., 2022; Muñoz-Mahamud et al., 2013; Shah et al., 2016; Van Den Kieboom et al., 2021).

Chevillotte et al. (2009) illustrated this problem clearly. Although inflammatory markers were frequently elevated in patients with PPFs, their positive predictive value for infection was poor (29 % for CRP, 21 % for ESR, and 18 % for WBC), warranting cautious interpretation of these parameters when a PPF and infection coexist. The high sensitivity but low specificity in this context has also been shown by Van Den Kieboom et al. (2021) when applying MSIS thresholds, promoting combined inflammatory markers to improve the diagnostic performance in iPPFs (Van Den Kieboom et al., 2021). These results demonstrate that serum markers cannot reliably distinguish infection from trauma-related inflammatory response. This is in line with current evidence in FRIs and PJIs. A meta-analysis of serum inflammatory markers in FRIs showed that inflammatory markers are insufficiently accurate to diagnose late FRI (Van Den Kieboom et al., 2018). These results were confirmed for PJIs by Sigmund et al. (2020). In summary serum inflammatory markers show limited diagnostic accuracy in iPPF for two opposite, timing-dependent reasons. Acutely, fracture and surgical trauma themselves trigger a systemic acute-phase response, elevating CRP, ESR, and WBC independent of infection. This is consistent with the observation of Zagra et al. (2026) that serum markers cannot reliably distinguish infection from trauma-related inflammation (Zagra et al., 2026). In late or chronic FRI, the problem shifts to sensitivity: low-grade, biofilm-associated infections caused by low-virulence organisms (e.g., coagulase-negative staphylococci) provoke only a weak systemic response, so markers may remain normal despite active infection – consistent with the FRI consensus definition, which treats serum markers as merely suggestive rather than confirmatory (Metsemakers et al., 2018). Serum markers are thus compromised in opposite directions depending on timing, reinforcing the preference for synovial fluid and microbiological criteria in diagnosing iPPF.

The diagnostic utility of synovial fluid WBC in the subgroup of iPPFs was analyzed in detail by Shah et al. (2016). Although their concern that fracture-related inflammation or hemarthrosis could falsely elevate these values, their results could demonstrate that synovial WBC count provides excellent sensitivity for diagnosing iPPF (with an overall area under the curve (AUC) of 84 %). They identified optimal cut-offs of 2707 WBC µ L^−1^ and 77 % polymorphonuclear cells (PMN), similar to the cut-offs of 3000 WBC µ L^−1^ and 80 % for the % PMNs used in the diagnostic workup of PJI (McNally et al., 2021). In contrast to this, Van Den Kieboom et al. (2021) identified substantially higher cut-off values (4552 WBC µ L^−1^, 79.5 % PMN) compared to PJI alone, suggesting higher thresholds and utilizing all serum and synovial markers in combination (Van Den Kieboom et al., 2021). Preston et al. (2015) analyzed preoperative synovial fluid nucleated cell count and found a positive predictive value (PPV) of only 18 %, indicating that fewer than one-fifth of patients with elevated cell count following fracture ultimately develop iPPF. However, with a sensitivity of 100 %, a normal nucleated cell count effectively excludes infection in PPFs. These tests offer several advantages, including rapid availability, low cost, objectivity, and the ability to be performed pre- or intraoperatively without specialized resources. However, in infected fractures without joint involvement, synovial markers may not be elevated as the joint itself is not affected.

Lazic et al. (2022) evaluated the reliability of calprotectin in the pre- or intraoperative diagnosis of iPPF (Lazic et al., 2022). Calprotectin demonstrated higher sensitivity than synovial WBC and PMN, while WBC and PMN showed high specificity. However, these results may be subject to bias as standard synovial analysis was not feasible in 10 cases, which may lead to an underestimation of the sensitivity of WBC and PMN in their study, involving only 14 cases with confirmed infection.

Two studies analyzed histopathological assessment, including intraoperative frozen sections (Luyckx et al., 2022; Muñoz-Mahamud et al., 2013). While, intraoperative frozen section histopathology is reliable for PJI diagnosis in non-traumatic revisions, its accuracy decreases markedly after a recent fracture due to trauma-induced PMN infiltration. Muñoz-Mahamud et al. (2013) found a 66.6 % false-positive rate for histology results when diagnosing infection in revision arthroplasty for Vancouver B2 periprosthetic fractures (100 % sensitivity, 55 % specificity) (Muñoz-Mahamud et al., 2013). In the study of Luyckx et al. (2022) the PPV was only 5.3 %. In the non-traumatic group, PPV was 80.8 %, with a negative predictive value (NPV) of 95.3 %. The authors suggested to set higher PMN cell count thresholds in the presence of recent trauma, although no specific cut-off values were proposed (Luyckx et al., 2022). This reinforces the notion that histopathology should be interpreted as a supportive, not decisive, diagnostic criterion in iPPFs.

Finally, Barberis et al. (2024) investigated the relevance of unexpected positive cultures. They found positive cultures in 6.8 % of the cases (15/159), while in seven patients two positive cultures were present for the same pathogens, meeting the ICM criteria for PJI. The authors also found a significant association of preoperative elevated CRP in culture-positive PPFs, although this must be considered in light of the poor specificity of CRP in the context of trauma (Barberis et al., 2024).

The reviewed diagnostic studies demonstrate that diagnosing infection in iPPFs relies on careful integration of clinical, laboratory, and intraoperative findings rather than on isolated parameters. Serum inflammatory markers are readily available and inexpensive but lack specificity in the presence of fracture-related inflammation. Synovial fluid analysis currently appears to provide the highest diagnostic accuracy when the prosthetic joint is involved, although optimal threshold values for iPPFs remain uncertain and may differ from those established for isolated PJIs. Microbiological examination of multiple deep tissue samples remains the diagnostic gold standard, as in both FRI and PJI, and should form the cornerstone of the microbiological work-up whenever infection is suspected. The interpretation of culture results, particularly unexpected positive cultures, remains crucial and must follow the established principles of musculoskeletal infection management such as the EBJIS definition criteria for PJI and the FRI Consensus definition (McNally et al., 2021; Metsemakers et al., 2018).

Histopathology provides valuable complementary information but should always be interpreted within the clinical context, particularly in the presence of recent trauma, where fracture-related inflammatory changes may reduce its specificity. Emerging biomarkers such as synovial calprotectin have shown promising preliminary results but require validation in larger prospective studies. Consequently, the diagnosis of iPPFs should rely on a multimodal approach integrating clinical findings with laboratory parameters, synovial fluid analysis, microbiological cultures from deep tissue samples, and histopathological assessment, rather than on any single diagnostic modality.

### 4.2 Reported treatment strategies

The main treatment modalities in iPPFs are DAIR, one-stage exchange, and two-stage exchange, and the most appropriate modality has to be selected after a diagnostic work-up in a MDT case-by-case discussion. Of the 24 treatment studies reviewed, staged approaches – predominantly two-stage procedures – were described in 83.3 % of the studies and 77.2 % of the reported patients. DAIR procedures were reported in few carefully selected cases (3 out of 162 patients) (Baker et al., 2012; Matsuno et al., 2023). Whereas Baker et al. (2012) described a case report of an early debridement within 15 d postoperatively in *Clostridium perfringens* infection, Matsuno et al. (2023) reported a DAIR in 2 out of 12 patients (multi-sensitive *Staphylococcus aureus* and culture-negative; timing of surgery not reported). Both studies reported complete infection eradication. However, three case reports reported failure of DAIR necessitating conversion to two-stage revision (Bonacker et al., 2018; Font-Vizcarra et al., 2014; Nasser et al., 2020). In PJIs, implant retention should only be performed under the following recommended conditions according to the EBJIS position paper: early infections (up to 3 weeks after index surgery), a stable prosthesis, a non-difficult-to-treat pathogen, and a vital soft tissue envelope (Sigmund et al., 2025). Correspondingly, a DAIR procedure should only be performed in selected FRI cases with a stable osteosynthetic construct, sufficient fracture reduction, and vital bone and soft tissues (Alt et al., 2024). One-stage exchange is typically reserved for selected patients with a well-characterized infection, favorable soft tissue conditions, and the possibility of achieving complete debridement and stable reconstruction in a single operation. Two-stage exchange is commonly indicated in complex infections characterized by chronicity, extensive bone or soft tissue compromise, difficult-to-treat pathogens, or uncertainty regarding infection eradication following a single procedure (Zimmerli et al., 2004). A variety of reconstructive techniques have been described to address the mechanical challenges associated with iPPFs. However, the searched literature is highly heterogeneous with regard to implant selection and reconstructive strategies, and most studies provide insufficient detail to allow meaningful comparison between specific implants or fixation constructs. Consequently, the present review focuses on overarching treatment concepts rather than individual implant choices.

### 4.3 Outcome of iPPFs

The eligible studies ( n=9 ) showed a median infection eradication and recurrence rate of 76.1 % and 22.3 %, respectively, which are slightly lower than those commonly reported for isolated PJI or FRI cases. Infection eradication rates are generally 80 %–90 % for both PJI and FRI with optimal surgical and antibiotic management, with recurrence rates ranging from 10 %–32 % depending on infection chronicity, surgical strategy, and follow-up duration (Buijs et al., 2022; Morgenstern et al., 2021; Otten et al., 2021). However, the lack of standardized outcome reporting hinders the comparability of the results (Reinert et al., 2025a). The considerable range of infection eradication rate (58.3 % to 91.7 %) and infection recurrence rate (8.3 % to 29.2 %) may derive from heterogeneous, predominantly small observational studies and case series and the absence of the above-mentioned standardized outcome reporting. Therefore, they should be interpreted as descriptive estimates rather than definitive benchmarks.

Furthermore, the majority of the studies (14 out of 24) reported that additional revision surgeries were needed to cure infection. The elevated incidence of secondary surgical intervention highlights the considerable morbidity associated with iPPFs and underscores the necessity for comprehensive treatment approaches.

Fracture healing outcomes were reported in only four out of nine eligible studies. The mean bone healing rate was 91.4 %, while two studies documented a 100 % (Keller et al., 2022; Konan et al., 2011), one study a 62.5 %, and one study a 91 % bone healing rate (Müller et al., 2019; Sassoon et al., 2014). In iPPFs fracture healing is (in most scenarios) an equally important outcome next to infection eradication and should therefore be assessed and recorded routinely.

In order to plan treatment more accurately the two different clinical scenarios of iPPFs have to be considered (scenario F-I vs. I-F). The reported DAIR procedures were all performed in scenario F-I, in which a stable implant is a mandatory precondition. In the scenario I-F (10/24 studies), a near-universal need for staged revision and often complex reconstructions was noted (Table 2). This consistent pattern highlights that scenario I-F represents a biologically more compromised situation, which explains the more aggressive and staged surgical approaches observed in the literature. In addition, joint involvement of the infection and the fracture pattern with or without involvement of the prosthesis have to be considered in treatment planning. Other key determinants include infection chronicity, implant stability, and the condition of the soft tissue envelope, all of which should be incorporated into future treatment algorithms for iPPFs.

Three studies have proposed a structured treatment algorithm. Müller et al. (2019) and Jaschke et al. (2025) described a three-step approach consisting of an initial stage with removal of the prosthesis and all foreign material (step 1), fracture stabilization (step 2), and a second stage reimplantation (step 3) (Jaschke et al., 2025; Müller et al., 2019). In the cohort of Müller et al. (2019) revision surgery was required in four cases (50 %), and fracture healing was observed in five cases (62.5 %), whereas infection eradication was finally achieved in all eight cases (Müller et al., 2019). Jaschke et al. (2025) reported in their cohort of 46 patients an infection eradication of 63 %, whereas in 12 patients an infection persisted, and in 5 patients a resection arthroplasty had to be performed (Jaschke et al., 2025). The study of Faggiani et al. (2025) furthermore presents a workflow guiding treatment strategies divided into four scenarios based on the following variables: joint involvement, fracture healing, and infection progression (Faggiani et al., 2025). However, the proposed treatment algorithms do not discriminate the two scenarios: I-F vs. F-I. This is an important consideration to address all sorts of iPPFs.

A concurrent systematic review by Zagra et al. (2026) reached convergent conclusions, reporting infection eradication rates of 70 %–80 % and reinfection of 6 %–28 % across 13 studies and 210 patients. However, several important methodological and conceptual differences distinguish this paper from the present scoping review. First, and most critically, Zagra et al. (2026) do not apply the F-I/I-F scenario framework: findings are organized by treatment modality rather than by the temporal and causal relationship between fracture and infection. This means it cannot address the scenario-specific treatment implications that are central to the present review. Second, our review is broader in scope, with 32 studies and 273 patients. Third, whereas Zagra et al. (2026) frame diagnostics exclusively within the PJI literature, we explicitly identify the dual applicability of both PJI (MSIS/ICM, EBJIS) and FRI consensus frameworks – a distinction that is essential for iPPFs as a hybrid entity. Together, these differences justify the present review's conceptual framework and underscore that no prior review has addressed iPPF from a scenario-based perspective.

### 4.4 Limitations and future directions

The current evidence base for iPPFs is limited by small sample sizes and substantial heterogeneity in infection definitions, treatment strategies, and outcome reporting. Consequently, the findings of this scoping review should be interpreted with caution. Consistent with the methodology of a scoping review, no formal risk-of-bias assessment or quality appraisal was performed. Nevertheless, the available literature consists predominantly of small retrospective observational studies, case series, and case reports, with at least half of the treatment studies including three or fewer patients. As a result, the reported infection eradication, recurrence, and bone healing rates should be regarded as descriptive estimates derived from a very low-certainty evidence base rather than definitive benchmarks of clinical outcome. Allocation of studies to the F-I or I-F scenario was performed to the best of our knowledge based on the information reported in each source study; however, residual misclassification bias cannot be excluded, particularly where the temporal sequence of fracture and infection was not explicitly stated. This limitation should be considered when interpreting scenario-specific treatment strategies and outcomes. This underpins the need to implement a standardized terminology, which is key to increasing evidence in the field. Data on fixation or reconstruction technique and on implant stability – both clinically important determinants of treatment strategy in iPPF – were inconsistently reported across the included studies and could not be systematically extracted or tabulated.

Another limitation is that we did not include a time limitation when infection occurred after PPF. The timing between fracture and infection – and vice versa – is clinically relevant, particularly for distinguishing early acute from late/chronic presentations, which carry different diagnostic and therapeutic implications. In addition, the fracture healing status at the time of infection diagnosis is equally relevant: an unhealed fracture presenting with infection requires different management from a healed fracture. These temporal and healing-status variables should be systematically documented in future iPPF cohorts.

Moreover, as antibiotic management and multi-disciplinary-team involvement represent an equally important pillar alongside surgery for proper infection management, this review does not adequately address this aspect due to insufficient data reported in the primary studies.

Furthermore, we limited our literature search to iPPFs around the knee and hip joint, and we did not differentiate between the affected bones. Due to limited data a further reasonable sub-analysis is not constructive. Also, by only including studies published in English, relevant non-English-language publications may be missed and acknowledged as a potential source of selection bias.

## 5 Conclusion

This scoping review demonstrates that the management of iPPFs remains highly challenging. The current evidence base is limited and consists predominantly of small, heterogeneous observational studies and case series, with substantial variability in diagnostic definitions, treatment strategies, and outcome reporting. As a result, meaningful comparisons between studies and the formulation of evidence-based treatment recommendations remain difficult. These findings highlight the urgent need for standardized diagnostic criteria, treatment algorithms, and outcome measures, as well as large prospective multicenter studies. Importantly, iPPFs should be recognized as a distinct hybrid pathology requiring the integrated application of both FRI and PJI principles. By introducing the F-I/I-F framework and combining established PJI and FRI diagnostic concepts, this review provides a novel, scenario-specific approach to the evaluation and management of this complex clinical entity and establishes a foundation for future research and treatment algorithms.

## Data Availability

The data that support the findings of this study are available from the corresponding author Mario Morgenstern upon reasonable request.

## Appendix A

**Table A1:** Overview of isolated pathogens and local antibiotics used in cement spacers.

Authors and year	Isolated pathogens	Antibiotics in spacer
Hao et al. (2023)	Methicillin-resistant *Staphylococcus aureus* (MRSA)	Vancomycin using Palacos R+G, Heraeus Medical GmbH cement
Konan et al. (2011)	*S. aureus*: 4, polymicrobial: 5, *Enterococcus*, *S. epidermidis*, 5× no organisms isolated	Not mentioned
Bottner et al. (2005)	Not mentioned	Gentamicin
Font-Vizcarra et al. (2014)	*Escherichia coli*, *Enterococcus faecium*	Clindamycin and Gentamycin
Sherman et al. (2008)	Methicillin-resistant *Staphylococcus epidermidis* (MRSE)	Vancomycin (2 g) and Tobramycin (4 packets, 2 g per packet)
Matsuno et al. (2023)	*Staphylococcus aureus*: 7 cases; Methicillin-resistant *Staphylococcus aureus* (MRSA): 4 cases; Methicillin-susceptible *Staphylococcus aureus* (MSSA): 3 cases; *Escherichia coli*: 2 cases; *Enterococcus faecalis*: 1 case; unknown or undetected: 2 cases	Not mentioned
Schwab et al. (2003)	Methicillin-resistant coagulase-negative *Staphylococcus*	Vancomycin (2 g) and either Tobramycin (2.4 g) or Gentamycin (2.4 g) per package of cement
Sassoon et al. (2014)	Not mentioned	Not mentioned
Nasser et al. (2020)	*Salmonella* Typhimurium	Gentamycin and Meropenem
Pang et al. (2015)	*Staphylococcus epidermidis*	Simplex P cement with Tobramycin
Maceroli et al. (2019)	Not mentioned	Not mentioned
Kamath et al. (2012)	Methicillin-resistant *Staphylococcus aureus* (MRSA) – *Klebsiella pneumoniae*	Vancomycin and Tobramycin
Bonacker et al. (2018)	Multidrug-resistant *Staphylococcus epidermidis* (MRSE)	Copal G + C 1×40 mg; Palacos R + G 2×40 mg with additional antibiotic-impregnated chains
Keller et al. (2022)	*Staphylococcus aureus*: 8 cases; *Pseudomonas* spp.: 2 cases; *Enterobacter* spp.: 2 cases; *Candida* spp.: 1 case; no organism isolated: 1 case	Not mentioned
Cabral (2012)	*Staphylococcus aureus*: 2 cases; *Staphylococcus epidermidis*: 1 case	Not mentioned
Kim et al. (2017)	β -streptococci	700 mg of Daptomycin and 200 mg of Dibekacin sulfate
Hagen et al. (2013)	Clostridium species	2.4 g of Tobramycin was mixed in the 80 mg packet of bone cement.
Karczewski et al. (2022)	Coagulase-negative staphylococci: 47 cases (comparison group), 5 cases (fracture group); *Cutibacterium* spp.: 17 cases (comparison group), 3 cases (fracture group); *Staphylococcus aureus*: 16 cases (comparison group), 2 cases (fracture group): *Enterococcus* spp.: 9 cases (comparison group), 2 cases (fracture group); *Streptococcus* spp.: 8 cases (comparison group), 1 case (fracture group); Gram-negative rods: 4 cases (comparison group), 2 cases (fracture group); *Candida* spp.: 1 case (comparison group), 2 cases (fracture group); Difficult to treat (DTT) microbes: 7 cases (comparison group); 4 case (fracture group)	Not mentioned

**Table A1:** Continued.

Authors and year	Isolated pathogens	Antibiotics in spacer
Baker et al. (2012)	Clostridium perfringens	Not mentioned
Müller et al. (2019)	CNS (Coagulase-negative staphylococci): 4 cases; *Cutibacterium*: 2 cases; *Staphylococcus aureus*: 2 cases; *Haemophilus*: 1 case; *Streptococcus anginosus*: 1 case; *Klebsiella oxytoca*: 1 case; *Corynebacterium* sp.: 1 case; *Enterococcus faecalis*: 1 case	Gentamycin (0.5 g) and Vancomycin (2 g)
Jaschke et al. (2025)	Pathogens divided into high-virulence and low-virulence: *Staphylococcus aureus*, streptococci, enterococci, Gram-negative bacteria, and *Candida* strains were considered to be high-virulence, whereas coagoulase-negative staphylococci strains (CNS), *Cutibacterium*, and anaerobes were considered to be low-virulence.	Not mentioned
Faggiani et al. (2025)	Not mentioned	Not mentioned
Duong et al. (2025)	MSSA: 8; polymicrobial:5	2 g of Vancomycin and 2 g of Meropenem per 40 g of cement
Kraus et al. (2025)	*E. coli*, *Acinetobacter baumannii*	Copal^®^ G + C with 1 g Gentamycin and 1 g Clindamycin
